# Effectiveness of a far‐infrared low‐temperature sauna program on geriatric syndrome and frailty in community‐dwelling older people

**DOI:** 10.1111/ggi.14003

**Published:** 2020-08-09

**Authors:** Masamitsu Sugie, Kazumasa Harada, Tetsuya Takahashi, Marina Nara, Hajime Fujimoto, Shunei Kyo, Hideki Ito

**Affiliations:** ^1^ Department of Geriatric Health Promotion Tokyo Metropolitan Geriatric Hospital and Institute of Gerontology Tokyo Japan; ^2^ Department of Cardiology Tokyo Metropolitan Geriatric Hospital and Institute of Gerontology Tokyo Japan; ^3^ Institute of Gerontology Tokyo Metropolitan Geriatric Hospital and Institute of Gerontology Tokyo Japan; ^4^ School of Health Science Juntendo University Tokyo Japan; ^5^ Japanese Association for Healthy Life Expectancy Tokyo Japan; ^6^ Department of Cardiac Surgery Tokyo Metropolitan Geriatric Hospital and Institute of Gerontology Tokyo Japan; ^7^ Department of Diabetes, Metabolism, and Endocrinology Tokyo Metropolitan Geriatric Hospital and Institute of Gerontology Tokyo Japan

**Keywords:** accumulation deficit model, far‐infrared low‐temperature sauna, frailty, geriatric syndrome, low fitness

## Abstract

**Aim:**

Although it is known that geriatric syndrome is associated with the development of frailty, it is not known whether an amelioration of geriatric syndrome also improves shared risk factors and frailty.

**Methods:**

In total, 67 community‐dwelling older people (79.6 ± 6.5 years, 49 women) participated in this study (41 were classified as pre‐frail and 26 as frail). We analyzed indices of physical frailty and cognitive depression, exercise tolerance and health‐related quality of life as frailty related indices, and the participants completed a questionnaire regarding common geriatric symptoms (cold extremities, leg edema, breathlessness, urinary incontinence, chronic headache, chronic pain, a sense of numbness, anorexia, constipation, insomnia and skin trouble) using numeric ratings. Frailty was evaluated using the Japanese version of the Cardiovascular Health Study (J‐CHS) criteria. The participants then underwent a far‐infrared low‐temperature sauna (FILTS) program twice a week for 3 months and the above parameters were reassessed.

**Results:**

After the FILTS program, there were significant differences in usual walking speed, peak oxygen uptake, Geriatric Depression Scale‐15, health‐related quality of life and the severity of several geriatric symptoms. Of the 67 participants, 18 showed improvements in their J‐CHS frailty score, 47 showed no change and two showed reductions. Linear regression analysis showed that the change in the numeric rating of the coldness of extremities (B = −0.105, *P* = 0.013) and the cumulative numeric rating for geriatric syndromes (B = 0.044, *P* < 0.001) were independent determinants of the change in the J‐CHS score.

**Conclusions:**

A 3‐month FILTS program ameliorates geriatric syndrome, the severity of frailty and frailty related indices in older Japanese people. **Geriatr Gerontol Int 2020; 20: 892–898**.

## Introduction

In our aging society, there is an urgent demand for measures to prevent frailty and disability in community‐dwelling older people. Frailty is a common clinical syndrome in older adults that carries a higher risk of poor health outcomes, including falls, disability, hospitalization and mortality.^1^, ^2^, ^3^, ^4^, ^5^ Frailty is a biological syndrome that can manifest in a number of ways, but can be defined as the presence of three or more of the following five components: unintentional weight loss, self‐reported exhaustion, weakness, slow walking speed and low physical activity.^2^, ^6^ Inouye *et al*. proposed a unifying conceptual model in which shared risk factors may lead to the development of geriatric syndromes; in turn these may lead to frailty, with feedback mechanisms increasing the incidence of shared risk factors and geriatric symptoms, ultimately leading to disability, dependence and death.^7^ However, it is not known whether an amelioration of the geriatric syndrome would interrupt the feedback mechanisms, thereby improving shared risk factors and frailty.

Common geriatric conditions include delirium, falls, frailty, dizziness, syncope and urinary incontinence,^7^ but we also frequently encounter problems with cold extremities, anorexia, constipation, chronic pain, breathlessness, urinary incontinence, chronic headache, insomnia, leg edema and skin problems, which may or may not be clinically significant, in elderly Japanese outpatients.

We hypothesized that far‐infrared low‐temperature saunas (FILTS) might be able to reduce the degree of frailty in the elderly population, thereby interrupting feedback mechanisms and ameliorating shared risk factors and the geriatric syndrome.

A previous study has shown that FILTS ameliorates: dyspnea associated with chronic heart failure (i.e., Waon therapy) and chronic obstructive pulmonary disease; exercise intolerance associated with chronic heart failure; pain associated with fibromyalgia, chronic fatigue syndrome; ulcers and other skin lesions associated with peripheral artery disease; constipation associated with ileus; mild depression; and anorexia associated with depression.^8^ Although these symptoms are associated with specific diseases, many are consistent with those reported in geriatric people.

Therefore, in the present study, we aimed to determine whether FILTS ameliorates features of the geriatric syndrome, and whether such improvement is associated with the amelioration of frailty and frailty related indices.

## Methods

### *Participants*

Sixty‐seven community‐dwelling older people (18 men and 49 women) living in the Tokyo metropolitan area participated in this study. Their mean age was 79.6 ± 6.5 years (range 66–93 years). None of the participants were hospitalized at the time, but all were being treated on an outpatient basis at the Tokyo Metropolitan Geriatric Hospital and Institute of Gerontology. The inclusion criteria were as follows: pre‐frailty or frailty, determined using the Japanese version of the Cardiovascular Health Study (J‐CHS) criteria^9^; lack of participation in an exercise training program^10^ to reduce frailty resulting from a physical condition; and cognitive function sufficient to communicate. The exclusion criteria were as follows: age <65 years; visual impairment; hearing impairment; robust status, according to the J‐CHS criteria^9^; musculoskeletal impairment that might interfere with the ability to participate in a physical examination; clinical instability; and dementia. Potential participants who were undertaking a similar sauna program, such as Waon therapy,^8^ were also excluded from the study.

The clinical characteristics of the participants are summarized in Table 1. We assessed the participants using physical and cognition/depression frailty indices, assessed health‐related quality of life (HRQOL) as a frailty related index, and completed a geriatric syndrome questionnaire using numeric rating scales for all the participants. The participants were permitted to continue taking their existing medication, rehabilitation folk remedies and/or supplements during the study.

**Table 1:** Participants' characteristics and comparisons of the characteristics between pre‐frail and frail participants

		All *n* = 67 (%)	Pre‐frail *n* = 41 (%)	Frail *n* = 26 (%)	*p*
Hypertension	−	32 (47.6)	21 (51.3)	11 (41.7)	0.458
	+	35 (52.4)	20 (48.7)	15 (58.3)	
Diabetes mellitus	−	50 (74.2)	30 (73.7)	20 (76.9)	0.908
	+	17 (25.8)	11 (26.3)	6 (23.1)	
Dyslipidemia	−	42 (62.9)	26 (63.2)	16 (62.5)	0.958
	+	25 (37.1)	15 (36.8)	10 (37.5)	
Atrial fibrillation	−	62 (91.9)	37 (89.5)	25 (95.8)	0.64
	+	5 (8.1)	4 (10.5)	1 (4.2)	
Coronary artery disease	−	53 (79.0)	32 (78.9)	21 (79.2)	0.984
	+	14 (21.0)	9 (21.1)	5 (20.8)	
Chronic heart failure	−	56 (83.9)	35 (84.2)	22 (83.3)	0.927
	+	11 (16.1)	6 (15.8)	4 (16.7)	
Previous cardiac surgery	−	61 (90.3)	37 (89.5)	24 (91.7)	0.776
	+	6 (9.7)	4 (10.5)	2 (8.3)	
Cerebral infarction	−	62 (91.9)	38 (92.1)	24 (91.7)	0.951
	+	5 (8.1)	3 (7.9)	2 (8.3)	
COPD	−	66 (98.4)	41 (100.0)	25 (95.8)	0.205
	+	1 (1.6)	0 (0.0)	1 (4.2)	
Chronic kidney disease	−	63 (93.7)	38 (92.3)	25 (95.8)	0.577
	+	4 (6.3)	3 (7.7)	1 (4.2)	
Arteriosclerosis obliterans	−	67 (100.0)	41 (100.0)	26 (100.0)	
	+	0 (0.0)	0 (0.0)	0 (0.0)	
Knee osteoarthritis	−	58 (87.3)	34 (82.1)	25 (95.8)	0.111
	+	9 (12.7)	7 (17.9)	1 (4.2)	
Osteoporosis	−	63 (93.5)	38 (92.1)	25 (95.8)	0.561
	+	4 (6.5)	3 (7.9)	1 (4.2)	
Cancer	−	64 (95.2)	41 (100.0)	24 (91.7)	0.07
	+	3 (4.8)	0 (0.0)	2 (8.3)	
Lumbar canal stenosis	−	58 (87.1)	35 (84.2)	24 (91.7)	0.394
	+	9 (12.9)	6 (15.8)	2 (8.3)	

COPD, chronic obstructive pulmonary disease. Comparisons were made using the chi‐squared test.

### *Criteria for the diagnosis of pre‐frailty and frailty*

Frailty and pre‐frailty were diagnosed using the Japanese version of the Cardiovascular Health Study (J‐CHS) criteria.^9^ These criteria include five domains: weight loss, slowness, weakness, exhaustion and low physical activity. The participants were classified as robust, pre‐frail or frail, as previously described.^10^

### *Geriatric syndrome questionnaire with numeric rating scale*

We created a questionnaire that aimed to grade the symptoms of geriatric syndrome. The symptoms that were assessed using this questionnaire were coldness of extremities, leg edema, breathlessness, urinary incontinence, chronic headache, chronic pain (e.g., knee pain or back pain), a sense of numbness, anorexia, constipation, insomnia and skin trouble, all of which are features of geriatric syndrome in outpatients. The questionnaire was used to record the presence or absence of each symptom, and to grade the severity of each using a numeric rating scale from 0 to 10, where 10 is very severe. The major symptoms of geriatric syndrome, falls and aspiration, were excluded from this questionnaire because these can be recorded as present or absent, but it is difficult to grade their severity using a numeric scale.

### *Far‐infrared low‐temperature sauna program*

The FILTS program used a far‐infrared dry sauna (Onkan‐rebalance OR‐1507; Digi‐Tech Corporation, Takaoka City, Toyama Prefecture, Japan) that was maintained at 60°C. Participants remained seated for 15 min, and then rested in a supine position, while covered with a warm blanket, for an additional 30 min. All the participants were weighed before and after each FILTS session and instructed to drink more water than their weight loss from perspiration. The FILTS program consisted of two sessions per week for 3 months, for a total of 24 sessions.

### *Evaluation of physical performance and other parameters*

Body composition was assessed using skeletal muscle mass index and body mass index. Physical performance was assessed using handgrip strength, timed up‐and‐go test and usual walking speed (UWS). Cognitive function and depressive symptoms were assessed using the Japanese version of the Montreal Cognitive Assessment (MoCA‐J)^11^ and the 15‐item Geriatric Depression Scale (GDS‐15).^12^ HRQOL^13^ was assessed using a physical component scale (PCS) and a mental component scale on Short Form‐8 (SF‐8). Finally, exercise tolerance was assessed using cardiopulmonary exercise testing. All of these assessments were evaluated as previously described.^10^, ^14^, ^15^, ^16^, ^17^

### *Statistical analysis*

A sample size of 54 participants was calculated for 80% power, α = 0.05, β = 0.2, and anticipated effect size = 0.35 (linear multiple regression: fixed model, *R* ^2^ deviation from zero; a priori: compute the required sample size – given α, power and effect size) using sample size software (G*Power 3.1.9.2, Heinrich‐Heine‐Universität Düsseldorf, Germany).

The chi‐squared test was used to compare the prevalence of age‐related disease(s) between pre‐frail and frail older people (Table 1). To compare the numeric ratings of geriatric syndrome and frailty related indices between baseline and the end of the 3‐month FILTS program, we performed Wilcoxon signed rank test in Table 2 and Student's *t*‐test in Table 3. Chi‐squared tests (McNemar's tests) were performed to compare the pre‐frail and frail groups at baseline and after the 3‐month FILTS program (Table 4). To determine whether frailty was affected by the presence of the geriatric syndrome, multiple linear regression analysis was used to predict the change in J‐CHS score, after adjustment for the changes in the numeric rating of coldness of extremities, leg edema, breathlessness while walking, urinary incontinence, chronic pain, skin trouble, cumulative numeric rating of geriatric syndrome, age and sex (Table 5). To compare each index of frailty between baseline and the end of the 3‐month FILTS program we performed chi‐squared tests (McNemar's test) (Table S1). All the statistical analyses were performed using the Statistical Package for the Social Sciences version 22 (IBM Japan, Tokyo, Japan), and the significance level was set at *P* = 0.05 for all the tests.

**Table 2:** Comparisons of the numeric ratings of symptoms of the geriatric syndrome before and after intervention

	Presence*n* (%)	Pre‐intervention*n* (%)	Post‐intervention*n* (%)	*P*
Coldness of extremities	38 (56.7)	3.0 (0.0–7.0)	2.0 (0.0–4.0)	0.009
Leg edema	32 (47.8)	0.0 (0.0–5.0)	0.0 (0.0–4.0)	0.049
Breathlessness	3 (4.5)	0.0 (0.0–0.0)	0.0 (0.0–0.0)	1.000
Breathlessness while walking	29 (43.3)	0.0 (0.0–5.0)	0.0 (0.0–2.0)	0.002
Urinary incontinence	7 (10.4)	0.0 (0.0–0.0)	0.0 (0.0–0.0)	0.034
Urinary incontinence while walking	27 (40.3)	0.0 (0.0–2.0)	0.0 (0.0–2.0)	0.258
Chronic headache	18 (26.9)	0.0 (0.0–1.0)	0.0 (0.0–1.0)	0.089
Chronic pain	55 (82.1)	6.0 (2.0–8.0)	4.0 (2.0–7.0)	0.002
A sense of numbness	30 (44.8)	0.0 (0.0–4.0)	0.0 (0.0–4.0)	0.596
Anorexia	29 (43.3)	0.0 (0.0–5.0)	0.0 (0.0–4.0)	0.838
Constipation	32 (47.8)	0.0 (0.0–4.0)	0.0 (0.0–3.0)	0.633
Insomnia	28 (41.8)	0.0 (0.0–4.0)	0.0 (0.0–4.3)	0.766
Skin trouble	38 (56.7)	2.0 (0.0–5.0)	0.0 (0.0–4.0)	0.034
Cumulative numeric rating scale geriatric syndromes score	–	25.0 (16.0–39.0)	20.0 (11.0–31.0)	<0.001

Comparisons were made using Wilcoxon signed rank test.

**Table 3:** Comparison of frailty related physical indices before and after the intervention

	Pre‐intervention	Post‐intervention	
	Mean ± SD	Mean ± SD	*P*
Body mass (kg)	51.3 ± 10.5	51.0 ± 10.2	0.20
Body mass index (kg/cm^2^)	22.3 ± 3.5	22.2 ± 3.3	0.41
Skeletal muscle mass index (kg/cm^2^)	6.0 ± 0.9	6.0 ± 0.9	0.37
Men	6.5 ± 1.1	6.5 ± 1.1	0.95
Women	5.8 ± 0.6	5.7 ± 0.7	0.67
Hand grip strength (kg)	17.6 ± 6.2	17.4 ± 6.3	0.60
Men	24.3 ± 8.6	22.1 ± 9.4	0.14
Women	14.0 ± 3.5	14.9 ± 3.3	0.57
Usual walking speed (m/s)	0.7 ± 0.2	0.9 ± 0.2	0.05
Timed up‐and‐go test (s)	10.5 ± 5.2	10.0 ± 5.0	0.14
Peak oxygen uptake/weight (mL/kg/min)	14.3 ± 4.5	15.3 ± 4.5	<0.001
Peak metabolic equivalent	4.1 ± 1.4	4.4 ± 1.4	0.08
Peak power (W)	54.6 ± 24.5	59.8 ± 26.1	<0.001
MOCA‐J	22.6 ± 4.9	22.3 ± 5.4	0.37
15‐item Geriatric Depression Scale	4.7 ± 3.4	4.1 ± 3.4	0.04
Physical component scale (SF‐8)	40.8 ± 8.1	42.7 ± 7.9	0.03
Mental component scale (SF‐8)	50.0 ± 8.3	51.3 ± 7.5	0.13

MoCA‐J, Japanese version of the Montreal Cognitive Assessment; SF‐8, Short‐Form Survey 8, Japanese version. Comparisons were made using Student's *t*‐test.

**Table 4:** Comparisons between pre‐frailty and frailty participants before and after intervention

		After the 3‐month intervention			
		Robust *n* (%)	Pre‐frail *n* (%)	Frail *n* (%)	Total *N* (%)
Pre‐frail	*n* (%)	7 (17.1)	32 (78.0)	2 (4.9)	41 (100)
		2.2	3.0	−4.8	
Frail	*n* (%)	0 (0.0)	11 (42.3)	15 (57.7)	26 (100)
		−2.2	−3.0	4.8	
Total	*n* (%)	7 (10.4)	43 (64.2)	17 (25.4)	67 (100)

Comparisons were made using the chi‐squared test. *P* < 0.05, φ = 0.50 (adjusted standardized residuals).

**Table 5:** Multiple regression analysis for the prediction of the change in the Japanese version of the Cardiovascular Health Study score

	B	β	*P*‐value	LCI	UCI
Model 1					
Change in the cumulative numeric ratingof geriatric syndrome	−0.334		0.011	−0.588	−0.079
	0.030	0.324	0.008	0.008	0.052
Model 2					
Change in the cumulative numeric ratingof geriatric syndrome	−0.359		0.005	−0.604	−0.114
	0.044	0.472	<0.001	0.020	0.067
Change in the numeric rating of coldness of extremities	−0.105	−0.323	0.013	−0.188	−0.023

*R* ^2^ = 0.190.

B, regression coefficient; LCI, lower 95% confidence interval; UCI, upper 95% confidence interval.

The analysis was adjusted for the change in numeric rating of coldness of extremities, leg edema, breathlessness while walking, urinary incontinence, chronic pain, skin trouble, age, sex and the change in the cumulative numeric rating of geriatric syndrome.

### *Ethical considerations*

This study was approved by the ethics committee of the Tokyo Metropolitan Geriatric Hospital and Institute of Gerontology (authorization number: 240301) and conformed with the principles outlined in the Declaration of Helsinki. All participants gave their written informed consent before data collection.

## Results

In total, 67 participants successfully completed the 3‐month FILTS program and no adverse events related to the program were recorded; 41 (61.2%) participants were classified as pre‐frail and 26 (38.8%) as frail. The pre‐frail and frail participants were of similar ages (78.9 ± 6.3 vs. 80.5 ± 6.7 years). The clinical characteristics of each group are summarized in Table 1; there were no significant differences between the pre‐frail and frail participants.

Table 2 shows the prevalence of each symptom and the change in the numeric rating of the geriatric syndrome during the 3‐month program. There were significant improvements in the scores for coldness of extremities, leg edema, breathlessness while walking, urinary incontinence, chronic pain and skin problems, and the cumulative numeric rating of geriatric syndrome also improved. Furthermore, the ratings for none of the symptoms worsened during the FILTS program.

Table 3 shows the changes in frailty related indices during the 3‐month FILTS program. Significant improvements occurred in UWS (m/s), peak VO_2_/weight (mL/kg/min), peak power (W), GDS‐15 score and PCS in SF‐8; none of the indices worsened during the program. Among the components of SF‐8, significant improvements occurred in physical function (42.0 vs. 45.0, *P* = 0.007), general health (46.3 vs. 48.4, *P* = 0.025) and emotional score (47.4 vs. 49.6, *P* = 0.021). There were no significant changes in physical score (43.5 vs. 45.7, *P* = 0.101), physical pain (43.9 vs. 44.2, *P* = 0.816), vitality (47.4 vs. 48.3, *P* = 0.207), social functioning (47.9 vs. 49.3, *P* = 0.150) or mental health (48.3 vs. 50.2, *P* = 0.054).

Table 4 shows the change in frailty status of the participants during the 3‐month program. Of the 41 pre‐frail participants, seven (17.1%) improved to become robust, 32 (78.0%) remained pre‐frail and two (4.9%) deteriorated to become frail. Of the 26 participants in the frailty group, 11 (42.3%) improved to become pre‐frail and 15 (57.7%) remained frail (McNemar's test, *P* < 0.05).

We compared each index of frailty (weight loss, slowness, weakness, exhaustion and low physical activity) before and after the FILTS program using the chi‐squared test (Table S1), and found significant differences in weight loss and physical activity.

Table 5 shows the results of multiple linear regression analysis that was conducted to predict the change in J‐CHS score during the program, adjusted for changes in the numeric rating of coldness of extremities, leg edema, breathlessness while walking, urinary incontinence, chronic pain, skin trouble and the cumulative numeric rating of geriatric syndrome, which incorporated conventional risk factors, in addition to age and sex. The linear regression model shows that the change in the coldness of extremities rating (B = −0.105, *P* = 0.013) and the cumulative numeric rating of geriatric syndrome (B = 0.044, *P* < 0.001) were independent determinants of the change in J‐CHS score (*R* ^2^ = 0.19, *P* = 0.001).

## Discussion

Our study used data from consecutively recruited pre‐frail and frail older outpatients. Patients had no one specific underlying condition and were regularly attending the Tokyo Metropolitan Geriatric Hospital as outpatients for the management of chronic diseases. The prevalences of pre‐frailty and frailty were 61.2% and 38.8%, respectively. A previous study reported the prevalences of robustness, pre‐frailty and frailty to be 30.3%, 59.8% and 9.9% in Japanese men, and 25.3%, 64.7% and 10.0% in Japanese women, respectively.^18^ Therefore, the prevalence of frailty in the participants in the present study was higher than that of the general population.

In total, 67 participants successfully completed the 3‐month FILTS program and experienced no adverse events, which demonstrates the safety of FILTS for community‐dwelling pre‐frail and frail outpatients with chronic disease (Table 1). Table 2 shows the prevalence of common symptoms comprising geriatric syndrome in this study. According to the Comprehensive Survey of Living Conditions by the Ministry of Health, Labor and Welfare, Japan,^19^ the prevalences of these symptoms are cold extremities (0.7%), leg edema (1.8%), breathlessness (2.0%), urinary incontinence (1.6%), chronic headache (5.2%), chronic pain (back pain (16.6%) and joint pain (9.7%), a sense of numbness (4.2%), anorexia (0.4%), constipation (2.3%), insomnia (2.0%) and skin problems (1.6%) in community‐dwelling older people. Therefore, the prevalences of these symptoms were high in the present sample of pre‐frail and frail older people. Table 2 also shows that the FILTS program was effective at reducing the severity of some of these symptoms (coldness of extremities, leg edema, breathlessness while walking, urinary incontinence, chronic pain and skin trouble) and the cumulative numeric rating scale score for geriatric syndromes. However, it must be noted that the participants also continued taking their existing medication, rehabilitation folk remedies and/or supplements during the study.

Table 3 shows that the participants had relatively poor physical function, mild cognitive impairment, low physical HRQOL and, in particular, low physical fitness (peak VO_2_/weight 14.3 ± 4.5 mL/kg/min and peak metabolic equivalent (METs) 4.1 ± 1.4 METs). Previously, we have reported that the peak VO_2_/weight and peak METs in robust, pre‐frail and frail individuals are 18.7 ± 4.0 mL/kg/min and 5.6 ± 1.0 METs, 16.7 ± 4.5 mL/kg/min and 5.2 ± 1.0 METs, and 14.7 ± 4.1 mL/kg/min and 4.4 ± 0.9 METs, respectively^10^, ^20^ In addition, it is well known that a peak VO_2_/weight ≤14 mL/kg/min is a hallmark of low physical fitness, and it is accepted as an indication for cardiac transplantation in patients with heart failure with severe left ventricular dysfunction.^21^

Table 3 also shows the effectiveness of the program with regard to UWS, peak VO_2_/weight, peak power, GDS and SF‐8 PCS. It ameliorated weight loss and low physical activity but not slowness (there was an improvement in UWS from 0.7 ± 0.2 to 0.9 ± 0.2 m/s [*P* < 0.05], but the cut‐off value of UWS for frailty is 1.0 m/s), weakness or exhaustion (Table S1).

In addition, we analyze gender‐specific analyses among UWS, peak VO_2_/weight, peak power, because these frailty related indices were expected gender differences. Significant differences were seen in UWS (0.74 ± 0.20 to 0.86 ± 0.20 m/s, *P* = 0.03), peak VO_2_/weight (14.6 ± 4.6 m/s to 15.6 ± 4.7 mL/kg/min, *P* = 0.003), peak power (52.5 ± 23.8 W to 56.8 ± 23.1 W, *P* = 0.004) among female, but not in male (UWS; 0.82 ± 0.3 to 0.79 ± 0.26 m/s, *P* = 0.52, peak VO_2_/weight; 13.7 ± 4.4 m/s to 14.7 ± 4.2 mL/kg/min, *P* = 0.11, peak power; 65.1 ± 27.2 to 73.0 ± 33.3 W, *P* = 0.07).

Table 4 shows that FILTS can ameliorate frailty. Of the 67 participants, 18 (26.9%) improved, 47 (70.1%) were unaffected and two (3.0%) worsened during the program. This might be the result of the FILTS program, but it might also have been the result of natural transitions. A previous study showed that individuals classified as pre‐frail were subsequently classified as robust, pre‐frail or frail, with prevalences of 23.1%, 58.2% and 18.2%, respectively, after 3.9 years, and those who were classified as frail were subsequently classified as robust, pre‐frail and frail, with prevalences of 3.3%, 40.3% and 54.5% after the same period of time had elapsed.^22^ However, the natural transitions between categories have not been characterized in the short term, such as during a 3‐month period.

We next used multiple linear regression analysis to determine whether the J‐CHS score improved because of the changes in numeric ratings for each of the symptoms, and found that the cumulative numeric rating for geriatric syndrome improved during the FILTS program. Table 5 shows that the changes in numeric rating for coldness of extremities and the cumulative numeric rating for geriatric syndrome are independent determinants of the change in J‐CHS score. This finding might indicate that FILTS improves frailty by ameliorating the geriatric syndrome. Given that the change in the numeric rating of coldness of extremities is an independent determinant of the change in J‐CHS score, FILTS may be a useful tool to ameliorate frailty; more generally, warming for older people with pre‐frailty or frailty may be beneficial. In addition, because the change in cumulative numeric rating of geriatric syndrome is an independent determinant of the change of J‐CHS score, FILTS might also improve frailty by ameliorating shared risk factors and the geriatric syndrome.

Mitnitski *et al*. first described the accumulation of deficits in older people in the Frailty Index, which incorporates the following in one index: symptoms; signs; diseases; disabilities; laboratory, radiographic and electrocardiographic abnormalities; and social characteristics.^23^ The results of the present study show that reducing the severity of symptoms of the geriatric syndrome is associated with an improvement in frailty score. However, the frailty score used in the present study depended on the phenotype of the frailty, and therefore might represent a subtype of accumulation deficit model. Nevertheless, the results of the present study suggest that measures aimed at reducing the severity of the geriatric syndrome are an important means of reducing frailty, and that FILTS might represent a suitable method of achieving this.

To date, changes in exercise and nutrition have been used to prevent or ameliorate frailty in older adults.^24^, ^25^ However, in our aging society, many older people cannot exercise because of poor physical function and low fitness. Therefore, FILTS might represent an alternative method of treating unfit older people with poor physical function.

The present study had several limitations. First, although we recruited clinical outpatients consecutively and non‐selectively, there may have been some bias. This could be because we excluded potential participants with acute or unstable diseases and those who regularly exercised. Second, our sample size was relatively small to describe the data of each gender, and third, there was no control group.

In conclusion, amelioration of the geriatric syndrome, with a 3‐month FILTS program, is associated with an improvement in frailty score and frailty related indices in a group of elderly Japanese people.

## Disclosure statement

The authors declare no conflict of interest.

## Author contributions

MS, TT, MN and KH made substantial contributions to the study design. MS, TT and MN contributed to the acquisition of data and performed the statistical analyses. KH, HF, SK and HI contributed to interpreting the data. MS, KH, TT and MN wrote the manuscript drafts. All the authors critically reviewed and contributed significantly to the intellectual content of the manuscript. All the authors agreed on the final content of the manuscript.

## Supporting information


**Table S1** Comparisons of the indices of frailty (weight loss, slowness, weakness, exhaustion, low physical activity) before and after the intervention using the chi‐squared test.
