# Supplementary material for: Effectiveness of a far‐infrared low‐temperature sauna program on geriatric syndrome and frailty in community‐dwelling older people
Source: Geriatr Gerontol Int. 2020 Aug 9;20(10):892–8. doi: 10.1111/ggi.14003 (PMC7590093; doi:10.1111/ggi.14003)
Supplement: Supplementary file 1 — Table S1 Comparisons of the indices of frailty (weight loss, slowness, weakness, exhaustion, low physical activity) before and after the intervention using the chi‐squared test. [file GGI-20-892-s001.docx]

**Supplemental table 1**

Comparisons of the indices of frailty before and after the intervention

|  |  | After the intervention | | |
| --- | --- | --- | --- | --- |
|  |  | Absent | Present | *p* |
| Weight loss | - | 44 (2.6) | 3 (−2.6) | < 0.05 |
|  | + | 14 (−2.6) | 6 (2.6) |  |
| Slowness | - | 32 (4.7) | 5 (−4.7) | n.s. |
|  | + | 9 (−4.7) | 21 (4.7) |  |
| Weakness | - | 18 (5.2) | 5 (−5.2) | n.s. |
|  | + | 6 (−5.2) | 38 (5.2) |  |
| Exhaustion | - | 31 (2.9) | 7 (−2.9) | n.s. |
|  | + | 14 (−2.9) | 15 (2.9) |  |
| Low physical activity | - | 39 (4.6) | 2 (−4.6) | < 0.05 |
|  | + | 12 (−4.6) | 14 (4.6) |  |

Comparisons were made using the chi-square test. *P*<0.05, φ = .50 (adjusted standardized residuals). n.s.: non-significant.
